# The Hunter Syndrome-Functional Outcomes for Clinical Understanding Scale (HS-FOCUS) Questionnaire: item reduction and further validation

**DOI:** 10.1007/s11136-014-0703-y

**Published:** 2014-05-08

**Authors:** Ingela Wiklund, Mireia Raluy-Callado, Wen-Hung Chen, Joseph Muenzer, Juanzhi Fang, David Whiteman

**Affiliations:** 1 Evidera, London, UK; 2 Evidera, Bethesda, MD USA; 3 Department of Pediatrics, University of North Carolina at Chapel Hill, Chapel Hill, NC USA; 4 Shire, Lexington, MA USA; 5 Shire, Eysins, Switzerland

**Keywords:** Hunter syndrome, Mucopolysaccharidosis type II, Lysosomal storage disease, Patient-reported outcomes, Hunter Syndrome-Functional Outcomes for Clinical Understanding Scale (HS-FOCUS)

## Abstract

**Purpose:**

The Hunter Syndrome-Functional Outcomes for Clinical Understanding Scale (HS-FOCUS) Questionnaire is a patient and parent-completed disease-specific instrument used in Hunter syndrome (mucopolysaccharidosis II), a rare paediatric progressive multi-systemic lysosomal storage disease. The objective of this study was to shorten the number of items of the Questionnaire to reduce response burden while maintaining its content validity.

**Methods:**

Data collected in a clinical trial were used. An iterative process helped identifying redundant or low performing items based on content validity and psychometric properties. Validation on the retained items was assessed using patients and parent’s responses in terms of reliability, validity and responsiveness.

**Results:**

The HS-FOCUS was completed by 49 patients and 84 parents. Items were mainly removed owing to high floor effects, high inter-item correlations (>0.80) or inadequate content. The shortened patient and parent versions (18 and 21 items) each contained five function domains. Internal consistency and test–retest reliability were >0.70 for most domains, except Breathing and School/work. Concurrent validity was demonstrated by significant correlations (>0.30) with similar concepts of previously validated measures. Significant differences were found in all domain scores across levels of disability.

**Conclusions:**

The shortened HS-FOCUS is a reliable, valid and responsive measure, where burden in answering the Questionnaire was reduced without compromising its validity.

## Background

Hunter syndrome, mucopolysaccharidosis II (MPS II), is a rare, X-linked, progressive, multi-systemic, lysosomal storage disorder caused by a deficiency of the enzyme iduronate-2-sulfatase [1] with an estimated incidence of 1 per 170,000 male births [2]. A wide spectrum of clinical disease occurs from attenuated to severe, with highly variable rates of progression and degree of organ involvement, resulting in significant impairment of patient’s function and quality of life [3].

In order to monitor disease progression and evaluate treatment in clinical trials, a MPS II-specific instrument, the Hunter Syndrome-Functional Outcomes for Clinical Understanding Scale (HS-FOCUS) Questionnaire, was developed by a group of experts led by Dr J Muenzer. Parent-completed and patient self-reported Questionnaires were created through the literature review and input from expert clinicians, parents and patients. Assessment of measurement properties of the HS-FOCUS was recently undertaken [4] and revealed that it was a valid and reliable instrument but could be further improved by reducing items that were redundant.

This communication is a follow-up to the previous publication [4] with the objective to reduce the number of items of the HS-FOCUS in order to have a shorter and more efficient instrument that also decreases the respondent burden.

## Methods

Data collected in a Phase II/III trial (NCT00069641) [5] as used in the validation study [4] were further analysed to reduce the items and re-assess the measurement properties of the shortened HS-FOCUS.

The original HS-FOCUS could be completed by parents/caregivers (68 items) or patients themselves (54 items). In both, items were grouped into six function domains: Walking/standing, Reach/grip, Sleeping, School/work, Activities and Breathing; and a satisfaction-with-function and a botheredness-with-function domains [4]. The item response scale ranged from 0 being ability to complete the activity ‘without ANY difficulty’ to 4 as ‘UNABLE to do so’. Average domain scores were computed using item responses if less than half of them were missing or ‘not applicable’.

Parents of patients of all ages responded to the parent-completed HS-FOCUS and patients aged ≥12 years to the self-reported version. The same participants also completed the following measures:

- *The Childhood Health Assessment Questionnaire (CHAQ)* A 30-item disease-specific instrument [6] that comprises eight domains: dressing, arising, eating, walking, reach, grip, hygiene and activities. Each domain is scored from 0 ‘without ANY difficulty’ to 3 ‘UNABLE to do’, and the average of the domain makes up the disability index score (DIS).
- *Health Utilities Index (HUI3)* A family of generic health profiles and preference-based systems with eight attributes: vision, hearing, speech, ambulation, dexterity, emotion, cognition and pain, with five to six levels per attribute [7]. It includes self-assessed and proxy-assessed forms. The scoring system provides utility (preference) scores on a scale where dead = 0 and perfect health = 1.
- *Childhood Health Questionnaire (CHQ*) A generic Questionnaire that measures 12 unique physical and psychosocial concepts in the children’s version CHQ-CF87 and 14 in the parent-completed CHQ-PF50: physical functioning, role/social–emotional and role/social–behavioural, role/social–physical, bodily pain, general behaviour, mental health, self-esteem, general health perceptions, change in health, parental impact–emotional, parental impact–time, family activities and family cohesion. Concept scores may also be combined to derive overall physical and psychosocial scores [8] from 0 to 100.

Statistical analysis of the parent-completed and patient self-reported responses were analysed separately using Stata/MP Ver.11.0 [9].

An iterative process was used to identify potentially redundant or poorly performing items using distributional characteristics of the HS-FOCUS item responses (mean, standard deviation, % missing and % at floor and ceiling). Each item identified was further reviewed and discussed individually and in the domain context. Input from two paediatric clinicians with long standing experience in treating MPS II was sought throughout the process.

Potentially redundant items were identified by any of the following criteria: high (≥60 %) percentage of missing responses suggesting the item was less relevant to MPS II or it was not clear; floor effects with ≥60 % responding ‘without ANY difficulty’ suggesting the item had a low impact on the majority of MPS II patients; high inter-item correlations (>0.80) suggesting item was redundant; low item-to-total scale correlation (<0.30) and poor clinical relevance. Items were examined by clinical experts for fit within the domain, who also ensured that no clinically relevant items were deleted.

Measurement properties of the shortened HS-FOCUS were then re-assessed. Internal consistency was assessed using Cronbach’s alpha, with *α* > 0.70 considered acceptable [10–12]. Test–retest reliability was considered acceptable when intra-class correlation coefficient (ICC) >0.70 among stable subjects defined as those whose CHAQ DIS score change did not exceed ±0.13 between baseline and week 18 [13]. Concurrent validity was evaluated with Spearman rank order correlations with CHAQ, CHQ and HUI3 [13]; correlations ≥0.30 between these measures were anticipated. Correlation with the original HS-FOCUS [4] was also calculated to support that content validity was maintained. Known-groups validity was determined using analysis of variance (ANOVA) to compare the mean of the shortened HS-FOCUS domain scores among three levels of disability: mild (CHAQ DIS ≤0.63); mild-moderate (CHAQ DIS >0.63 to ≤1.75) and moderate (CHAQ DIS >1.75) [14]. The extent to which the shortened HS-FOCUS could detect change in patients’ health status was assessed by comparing the mean domain scores in patients who showed improvement measured by a CHAQ DIS decrease ≥0.13 [14] versus those who did not.

## Results

Altogether, 49 MPS II patients 12 years and older and 84 parents completed the HS-FOCUS. The average age when diagnosed was 5 years (SD 4.75; median 4; P10–P90: 0–13). Patients’ demographic and clinical characteristics have been previously described in the HS-FOCUS validation study [4].

Table 1 shows the number of items retained for each HS-FOCUS domains and the reasons for item removal (the full content of the items is not shown because HS-FOCUS is proprietary). One of the items in the Sleeping domain was moved to Breathing, and two items in the Activities domain were moved to Grip/reach. Subsequently, the Sleeping domain were removed from the HS-FOCUS because after moving one item to breathing the remaining items were considered inadequate, and also because there are other well-established sleep measures (e.g., Tayside Children’s Sleep Questionnaire [15] or the Infant Sleep Questionnaire (ISQ) [16] ) that could be used to supplement the HS-FOCUS.

**Table 1:** Item reduction in the patient and parent versions of the HS-FOCUS

Domains	Items retained in the patient version	Items retained in the parent version	Items retained	Item removed (reasons for deletion)
Walking/standing	4 out of 10	5 out of 10	Walk and stand without getting tired; able to step on a stool or walk up stairs	Walk flat feet; stand on one foot; stand straight against a wall, etc. (poor content validity; duplication with other items and not cross-culturally relevant)
Grip/reach	4 out of 12 (+2)	6 out of 12 (+2)	Touch top of head; put on shoes; button a shirt; catch a balls, turn book pages, etc.	Pick up a hamburger with thumb and fingers; clap hands together; straight out arms to the side, etc. (low correlation with other items in the domain; high floor effects; poor content validity)
Sleeping	0 out of 4	1 out of 5 that was moved to the BREATHING domain due to closeness in clinical content	None (propose to be assessed by other well-established sleep measures)	Fall asleep within 20 minutes; sleep through night; feel rested after sleep, etc. (inappropriate domain for the clinical group)
School/work	2 out of 3	2 out of 3	Able to work or attend school; able to complete assignments of tasks	Pay attention in school/work (poor content validity)
Activities	5 out of 6: 3 in this domain; 2 more were moved to the GRIP/REACH domain due to closeness in clinical content	4 out of 6: 2 in this domain; plus 2 more were moved to the GRIP/REACH domain due to closeness in clinical content	Participate in physical activities or play with others; play video games	Go out with friends (poor content validity)
Breathing	3 out of 6	3 out of 6 (+1)	Breath without noise; talk or do activities without becoming short of breath	Blow out a candle; breath easily through nose; etc. (high floor effects)
Satisfaction-with-function	0 out of 6	Your child: 0 out of 6 You: 0 out of 6	None	Poor clinical relevance
Botheredness-with-function	0 out of 7	Your child: 0 out of 7 You: 0 out of 7	None	Poor clinical relevance

Consequently, both patient-completed (18 items) and parent-completed (21 items) Questionnaires of the shortened HS-FOCUS each contained five functional domains ranging from 0 to 3 (authors’ note: The HS-FOCUS is currently not publicly available; plan for sharing is under consideration, please contact Shire for more information). Table 2 shows the correlation between the shortened version and the original HS-FOCUS was above 0.85 except for the parent version’s Activities score (*r* = 0.75), supporting that content validity was maintained.

**Table 2:** Correlation coefficient between the original and shortened HS-FOCUS domains scores at baseline

	Patients’ responses		Parent’s responses	
	Spearman correlation coefficient	*N*	Spearman correlation coefficient	*N*
Walking/standing	0.94	49	0.97	84
Grip/reach	0.94	49	0.91	84
School/work	0.94	44	0.87	79
Activities	0.88	48	0.75	84
Breathing	0.86	45	0.85	79
Overall function score	0.95	49	0.89	84

Except for the Breathing domain in the patient’s sample, the internal consistency reliability for all five domains in the shortened HS-FOCUS was very good, with *α* ranging from 0.67 to 0.90 and 0.76 to 0.87 in the patients and parents’ sample, respectively. Overall, complete data on the HS-FOCUS and the CHAQ at baseline and week 18 was available for 82 parents and 48 patients 12 years old and above, from which 30 and 14 were rated stable, respectively. Good test–retest reliability was found with ICC ranging from 0.72 to 0.97 using patient responses and from 0.64 to 0.90 with parent responses. Concurrent validity indicated domains were consistently correlated to other instruments in the expected way. Moderate (>0.3) to high (>0.6) Spearman correlations were found between all patient self-reported domains and most CHAQ Scales; moderate to high correlations were found with the CHQ physical function, role/social physical, bodily pain and self-esteem scores, but low correlation (<0.3) with behaviour-related items, general health perception, change in health and family cohesion. Moderate to high correlations were found with the HUI3 utility scores: hearing, ambulation, dexterity, pain and overall HRQL utility. A similar correlation structure was found for the parent-completed responses (data not shown).

Excellent known-groups validity was shown with statistically significant differences (*P* < 0.01) in the ANOVA for most shortened HS-FOCUS domains between groups (Table 3). Results also indicated ability to detect changes in the patient’s condition with a statistically significant difference (*P* < 0.01) in score changes between the two groups in all five domains (Table 4).

**Table 3:** Known-groups validity for the shortened HS-FOCUS domains

	Mild (CHAQ DIS ≤0.63) *N* Mean (SD)	Mild/Moderate (CHAQ DIS >0.63 to ≤1.75) *N* Mean (SD)	Moderate (CHAQ DIS >1.75) *N* Mean (SD)	F value (*P* value)
Patients’ responses				
Walking/standing	5 0.55 (1.10)	27 0.83 (0.62)	17 1.81 (0.77)	11.28 (*P* < 0.001)
Grip/reach	5 0.37 (0.25)	27 0.94 (0.38)	17 1.82 (0.50)	33.70 (*P* < 0.001)
School/work	5 0.30 (0.67)	26 0.31 (0.65)	13 1.00 (1.02)	3.64 (*P* = 0.040)
Activities	5 0.40 (0.65)	26 0.54 (0.53)	17 1.68 (1.04)	12.80 (*P* < 0.001)
Breathing	4 0.50 (0.43)	27 0.68 (0.56)	14 1.21 (0.70)	4.36 (*P* = 0.020)
Parents’ responses				
Walking/standing	7 0.23 (0.62)	32 0.51 (0.39)	44 1.40 (0.72)	25.30 (*P* < 0.001)
Grip/reach	7 0.34 (0.39)	32 1.12 (0.50)	44 1.76 (0.50)	33.29 (*P* < 0.001)
School/work	7 0.29 (0.76)	31 0.34 (0.44)	40 0.94 (0.94)	6.20 (*P* < 0.001)
Activities	7 0.29 (0.57)	32 0.27 (0.40)	44 1.23 (0.87)	19.39 (*P* < 0.001)
Breathing	7 0.68 (0.70)	30 0.99 (0.67)	44 1.44 (0.72)	5.59 (*P* = 0.010)

**Table 4:** Ability of the shortened HS-FOCUS domains to detect change

HS-FOCUS domains	Patients/parents with CHAQ DIS score decrease ≥0.13		Patients/parents with CHAQ DIS score decrease <0.13		*P* value^c^
	*N*	Mean (SD)	*N*	Mean (SD)	
Patients’ responses^a^					
Walking/standing	23	−0.48 (0.64)	25	0.09 (0.56)	0.0002
Grip/reach	23	−0.38 (0.47)	25	−0.04 (0.51)	0.0025
School/work	19	−0.24 (0.42)	23	−0.07 (0.71)	<0.0001
Activities	22	−0.27 (0.61)	24	0.04 (0.97)	0.0019
Breathing	20	−0.50 (0.52)	22	0.09 (0.75)	<0.0001
Parents’ responses^b^					
Walking/standing	29	−0.50 (0.56)	49	−0.11 (0.54)	<0.0001
Grip/reach	29	−0.36 (0.51)	49	0.02 (0.45)	<0.0001
School/work	26	−0.27 (0.51)	45	0.04 (0.74)	<0.0001
Activities	29	−0.34 (0.61)	49	−0.12 (0.97)	<0.0001
Breathing	28	−0.55 (0.57)	46	−0.14 (0.67)	<0.0001

^a^Mean score change from baseline to week 53 as rated by patient

^b^Mean score change from baseline to week 53 as rated by parent

^c^ANCOVA model: HS-FOCUS domain score change = patient’s age + HS-FOCUS baseline score + CHAQ DIS patient/parent status

## Conclusions

The shortened HS-FOCUS retains the items that assess the most critical functions of patients with MPS II, and has shown to be a reliable, valid and responsive tool. For rare and debilitating diseases such as MPS II, good compliance with any instrument used to monitor disease progression over time and evaluate treatment benefits in clinical trials is essential. At the same time, it is important to reduce the respondent burden, especially in paediatric populations. Respondent fatigue when completing the Questionnaire may affect the validity of the scores [17]; therefore, reducing the length of the Questionnaire is expected to have a positive effect.

The current study is limited by the small sample size overall and by unbalanced groups when assessing known group validity; the latter may cause violation to the homogeneity of variance assumption of ANOVA. In addition, the use of mean imputation has the risk of creating a biased score; scoring should be further studied. Analyses were based on data of the original HS-FOCUS, and the complete validation of the shortened HS-FOCUS will be conducted in the future as collection of more data is ongoing.
